# Hematologic Derangements among Children with Unoperated Cyanotic Congenital Heart Disease in Ethiopia

**DOI:** 10.4314/ejhs.v33i6.5

**Published:** 2023-11

**Authors:** Selamawit Alemseged, Endale Tefera

**Affiliations:** 1 Department of Pediatrics and Child Health, Saint Peter Specialized Hospital, Addis Ababa, Ethiopia; 2 Department of Pediatrics and Adolescent Health, Faculty of Medicine, University of Botswana, Gaborone, Botswana

**Keywords:** Cyanotic congenital heart disease, hematologic derangements, coagulopathies, Unoperated heart defects

## Abstract

**Background:**

Surgical treatment has transformed the course and outcome of congenital heart defects in high-income countries, but children with congenital heart diseases in sub-Saharan Africa, where access to cardiac surgery is limited, often experience the natural course of untreated lesions and their complications. The objective of this study was to determine the prevalence of hematologic derangements among Ethiopian children with unoperated cyanoticcongenital heart diseases, to identify factors associated with coagulopathy in this population, and to describe how these complications are managed in this setting.

**Methods:**

In this single-center cross-sectional study, we prospectively collected clinical and demographic data from children (<18 years) with cyanotic congenital heart diseases. Blood samples were collected to measure hematologic parameters. Polycythemia was defined as hematocrit >50% and thrombocytopenia as <150,000 per microliter.

**Results:**

Among 70 children recruited, the overall prevalence of polycythemia and thrombocytopenia was 63% (n=44) and 26% (n=18), respectively. On multivariate logistic regression analysis, hematocrit ≥65% (p-value=.024), and oxygen saturation <85% (p-value=.018) were independently associated with moderate or severe thrombocytopenia. Thirty-one (44%) patients had undergone therapeutic phlebotomy, and 84% (26/31) of these patients received iron supplementation.

**Conclusion:**

We report a high prevalence of polycythemia and thrombocytopenia in Ethiopian children with untreated cyanotic congenital heart diseases. There was variable implementation of iron supplementation and therapeutic phlebotomy, highlighting the need to optimize supportive management strategies in this population to mitigate the risk of life-threatening complications.

## Introduction

Congenital heart defects (CHDs) are the most common human birth defects with an estimated incidence of 9-14 cases per 1000 live births ((1,2)). Although surgical and percutaneous treatments have dramatically changed CHD course and outcome among children in high-income settings, children in low- and middle-income countries (LMICs) in sub-Saharan Africa are less likely to access these treatments and often experience the natural course of untreated lesions. In particular, untreated cyanotic and complex CHDs are associated with several complications which can disrupt quality of life from an early age.

Among the many kinds of possible CHD complications, hematologic derangements and coagulopathies are the most important and can be life-threatening. Secondary polycythemia and increased blood viscosity, impaired blood flow dynamics and red cell deformation, iron deficiency anemia precipitated by polycythemia, thrombocytopenia, and coagulopathies are all well-described in patients with cyanotic CHD ((3–11)). Endothelial dysfunction, vascular dysfunction, and altered structure may also play a role in increasing the risk of cardiopulmonary insufficiency as well as thromboembolic events in children with CHD (12,13). Other complications include severe malnutrition and failure to thrive which can predispose children with cyanotic CHD to recurrent infections ((14,15)).

Some of the known conservative care modalities that can improve the quality of life in patients with cyanotic CHD, such as administration of iron and cautious phlebotomy in those with extremely high hematocrit, are not uniformly optimized in most sub-Saharan African settings. This study sought to determine the prevalence of hematological derangements among a group of children and adolescents with untreated cyanotic CHD in Ethiopia, to identify factors associated with coagulopathy, and to describe their management in this setting.

## Methods

This was a single-center cross-sectional study conducted at the Pediatric Cardiac Clinic of the Addis Ababa University Hospital (Tikur Anbessa Hospital) which receives approximately 200 referrals for cyanotic CHD annually from all regions of Ethiopia. Children and adolescents up to 18 years of age whose diagnosis of cyanotic CHD confirmed by echocardiography, were recruited between June 2016 and May 2017. Patients were excluded if they had pre-existing unrelated hematological disorders or coagulopathies. Written informed consent was obtained from parents/guardians and verbal child assent was also obtained from the patients for drawing blood samples (signed written informed consent forms are available along with the data). Ethical approval was obtained from the institutional review board.

**Data collection**: Using a standardized and pre-piloted data abstraction form, we collected: 1) Demographic data: age, gender, and residence; 2) Anthropometric data: weight, height, weight-for-age, height-for-age, and Body mass index; 3) Clinical data: diagnostic echocardiography, age at the time of diagnosis, oxygen saturation, iron supplementation, propranolol treatment, history of previous phlebotomy for polycythemia, clinical symptoms and signs attributable to hematologic derangements including chronic headache, dizziness, syncopal attacks, irritability, visual disturbance, tinnitus, paresthesia, myalgia, easy bruising/mucosal bleeding, and diagnosis of cerebrovascular accidents (CVA) or brain abscess. After written consent/assent was secured from the parents/guardians and patients; blood was drawn for determination of hemoglobin, hematocrit, red blood cell indices including mean corpuscular volume (MCV), mean corpuscular hemoglobin (MCH), and mean corpuscular hemoglobin concentration (MCHC) and red cell distribution width (RDW); platelet count; white blood cell count; coagulation profile (prothrombin time [PT], partial thromboplastin time [PTT], and international normalized ratio [INR]); serum creatinine and uric acid. We did not have a facility for the determination of serum ferritin, total iron-binding capacity, or transferrin.

**Case definitions**: For the purpose of this study, polycythemia was defined as hematocrit of >50% (16). However, there is no universally agreed cutoff point for diagnosis of polycythemia in patients with cyanotic congenital heart diseases. Furthermore, normal hematocrit may be age- and sex-dependent. Polycythemia requiring therapeutic phlebotomy (partial exchange transfusion) was defined as hematocrit >65%, according to the American Heart Association recommendation (17). In our setting, phlebotomy is generally indicated in patients presenting with hematocrit ≥65% with symptoms attributable to hyperviscosity, or hematocrit of ≥70% irrespective of symptomatology. Thrombocytopenia was defined as platelet count of <150,000 per microliter of blood (18), and sub-classified as mild (101,000-140,000/microliter), moderate (51,000-100,000/microliter), severe (21,000-50,000/microliter) and very severe (≥20,000). High red cell distribution width (RDW) was defined as > 15.5% (19).

Anthropometric measurements were collected and described for each participant. Being moderately underweight was defined as having weight-for-age between -3 and -2 standard deviations (SD) or BMI <18.5 kg/m^2^; children with weight-for-age <-3SD or with BMI <16.5 kg/m^2^ were considered severely underweight. Moderate stunting was defined as height-for-age between -3SD & -2SD; severe stunting was defined as height-for-age <-3SD.

**Sampling**: The sample size was calculated based on the expected prevalence of hematological derangements (at least one deranged parameter) in patients with cyanotic congenital heart diseases, which was assumed to be 50% (0.5). We used a margin of error of 10% instead of the usual 5% due to limited budget for laboratory costs. Using the single sample population proportion formula and applying the finite population correction factor, the calculated sample size was 66 to achieve 80% power and accurately estimate the prevalence of hematological abnormalities within 10% margin of error. We recruited consecutive patients until our sample size was achieved.

**Statistical methods**: Data were analyzed using Statistical Package for Social Sciences (version 27). Continuous variables were described using medians and interquartile ranges or means and standard deviations based on the distribution of the variables. Categorical variables were summarized using frequencies. Statistical associations were tested using the Mann-Whitney U test for continuous variables and the X^2^ test for categorical variables. Possible predictors for thrombocytopenia were entered into multivariate logistic regression after assessing the basic assumptions for logistic regression are not grossly violated (20). A p-value of 0.05 was taken as a level of statistical significance. GGplot2 package in RStudio software version 2021.09.1+372 was used to prepare a scatter plot of hematocrit vs. platelet count ((21)).

## Results

A total of 70 patients were recruited for the study. The median age was 4.9 years (interquartile range of 2.6 – 8.8 years) with the youngest and oldest ages being 3 months and 18 years, respectively. Forty-five (64.3%) patients were females. Most patients (55.7%) were from the capital city, Addis Ababa. Tetralogy of Fallot (TOF) accounted for the majority (62.8%) of the cardiac diagnoses. The demographic, anthropometric, and clinical characteristics of the patients are displayed in Table 1. Regarding anthropometric parameters, 20 (29%) of the patients were moderately underweight, and 22 (31.4%) patients were severely underweight. Twenty-three (33%) of the patients had moderate stunting and 10 (14.3%) had severe stunting. BMI was also moderately or severely low in nearly half of the patients (Table 1).

**Table 1:** Demographic and clinical characteristics of children and adolescents with cyanotic congenital heart diseases

Variables		Statistics
Gender – Female, n (%)		45 (64.3)
Age (years), median (IQR)		4.9 (2.6 – 8.8)
Weight (kg), median (IQR)		17.0 (10.3 – 20.0)
Weight-for-age classification		
Normal (>-2SD), n (%)		28 (40.0)
Moderate underweight (between -3SD & -2SD), n (%)		20 (28.6)
Severe underweight (<-3SD), n (%)		22 (31.4)
Height/length (cm), median (IQR)		98.0 (82.3 – 120.0)
Height-for-age classification		
Normal (>-2SD), n (%)		37 (52.9)
Moderate stunting (between -3SD & -2SD), n (%)		23 (32.9)
Severe stunting (<-3SD), n (%)		10 (14.3)
Body Mass Index (BMI) in Kg/m^2^, median (IQR)		14.0 (13.0 – 16.0)
Weight-for-length/height for <2-year-olds and BMI for 2-18-year-olds		
Normal (>-2SD), n (%)		36 (51.4)
Moderate underweight (between -3SD & -2SD), n (%)		13 (18.6)
Severe underweight (<-3SD), n (%)		21 (30.0)
Residence		
Addis Ababa, n (%)		31 (44.3)
Out of Addis Ababa, n (%)		39 (55.7)
Oxygen saturation (%), mean (IQR)		74.0 (67.0 – 85.0)
	Tetralogy of Fallot and its variants	44 (62.8)
Types of cyanotic congenital heart diseases	Transposition of the great arteries	6 (8.6)
	Double Outlet Right Ventricle (DORV)	6 (8.6)
	Tricuspid atresia	4 (5.7)
	Ebstein's anomaly	4 (5.7)
	Truncus arteriosus	3 (4.3)
	Others	3 (4.3)

The overall prevalence of polycythemia was 63% (44/70), with 17 patients (24.3%) having a hematocrit of ≥65% and 9 patients (12.9%) having hematocrit between 70% and 78%. The prevalence of thrombocytopenia was 26% (18/70); thrombocytopenia was classified as mild in 7 patients; moderate in 7 patients; severe in 3 patients; and very severe in 1 patient. RDW was greater than 15% in 52 (74.3%) of the patients with the highest value being 54%. With respect to coagulation profiles (n=59), 25 patients had elevated INR with corresponding high PT and PTT. Of these 25 patients, 21 had INR between 1.4 – 2.0; two patients had INR between 2.0 – 2.9; and two had INR ≥3.0. The details of hematological, coagulation, and other laboratory findings are shown in Table 2.

**Table 2:** Hematological, coagulation and other laboratory profiles of 70 children with unoperated cyanotic congenital heart diseases

Variables	Median (IQR)
Hemoglobin (gm/dl)	18.7 (14.4 – 21.3)
Hematocrit (%)	56.6 (42.2 – 63.5)
Mean Corpuscular Volume (MCV) in femtoliters (fl)	82.4 (74.3 – 87.5)
Mean Corpuscular Hemoglobin (MCH) in picograms per cell	27.7 (23.1 – 29.4)
Mean Corpuscular Hemoglobin Concentration (MCHC) in g/dl	32.8 (30.9 – 33.9)
Red Cell Distribution Width (RDW) in %	19.9 (15.4 – 25.0)
Platelets per microliter of blood	228,500 (141,000 – 289,000)
White Blood Cell (WBC) count, cells per cubic millimeter of blood	7590 (5750 – 9905)
Prothrombin time in seconds	17.8 (16.1 – 22.2)
Partial Thromboplastin Time (PTT) in seconds	45.2 (38.1 – 66.1)
International Normalized ratio (INR)	1.32 (1.19 – 1.61)
Blood Urea Nitrogen (BUN)	18.0 (15.0 – 23.0)
Creatinine	6.0 (5.0 – 7.0)
Uric acid	3.4 (3.0 – 4.0)

Two patients with polycythemia (2.9%) had a history of CVA. These were a 4-year-old girl and a 9-year-old boy who both had TOF and failure to thrive and presented to the referral center for the first time with CVA. In retrospect, both patients had significant symptoms that could have been attributable to hyperviscosity, including headache, dizziness, episodes of fainting, and visual disturbances. Five patients (7.1%) reported having easy bruising; fourteen patients (20%) reported having either recurrent epistaxis or gum bleeding or both. Other non-specific symptoms reported by those who can report subjective symptoms included chronic headache, dizziness, fainting, blurred vision, and tinnitus. Forty-eight (68.6%) patients received iron supplementation for a variable length of time. Thirty-one (44.3%) patients had undergone one or more rounds of therapeutic phlebotomy; of the 31 patients who had therapeutic phlebotomy, 5 patients were not started on iron supplementation.

**Figure 1:**
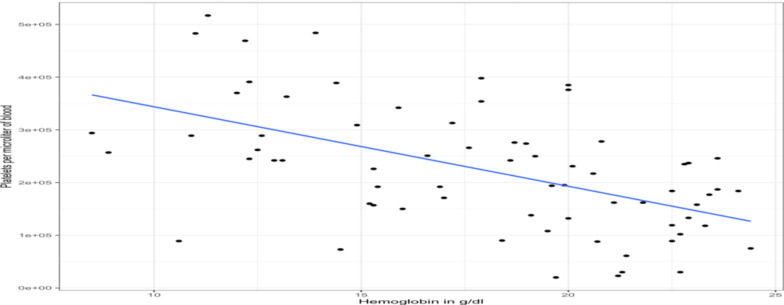
Scatter plot of hemoglobin vs. platelet count, showing negative correlation

Platelet count and hematocrit levels showed a negative correlation with a correlation coefficient of -0.57 (p-value <.001) (Figure 1). On unadjusted crude analysis, age ≥10 years, hematocrit ≥65%, RDW>15%, oxygen saturation <85%, history of one or more episodes of hypercyanotic spells, and one or more rounds of phlebotomies were significantly associated with a moderate or higher degree of thrombocytopenia (≤100,000/microliter of blood). However, on multivariate logistic regression analysis, only hematocrit ≥65% (p-value, .024), and oxygen saturation <85% (p-value, .018) were independently associated.

With respect to physiology of pulmonary blood flow, 52(74.2%) patients had cyanotic lesions with decreased pulmonary blood flow and 18 (25.8%) patients had lesions with increased pulmonary blood flow. Anthropometric and hematological profiles were not significantly different between the two groups except for MCH, MCHC, and RDW (Table 3).

**Table 3:** Anthropometric, haematological and coagulation profiles comparing patients with cyanotic congenital heart disease by pulmonary blood flow physiology

Variables	Decreased pulmonary blood flow group (n=52)	Increased pulmonary blood flow group (n=18)	p-value
Age in years (mean ±SD)	6.4 (4.5)	5.3 (4.4)	.467
Weight in kg (mean ±SD)	17.6 (9.9)	15.0 (11.7)	.408
Height in cm (mean ±SD)	107.0 (27.0)	97.3 (35.2)	.293
Oxygen saturation (%)	75.6 (11.9)	67.8 (15.0)	.054
Haemoglobin (g/dl)	18.2 (4.0)	16.6 (4.9)	.105
Haematocrit (%)	55.1 (12.3)	52.6 (13.4)	.487
Mean Corpuscular Volume (MCV) in femtoliters (fl)	81.9 (9.8)	77.5 (11.4)	.154
Mean Corpuscular Hemoglobin (MCH) in picograms per cell	27.4 (4.5)	24.0 (4.7)	.013
Mean Corpuscular Hemoglobin	32.7 (4.5)	30.9 (2.8)	.023
Concentration (MCHC) in g/dl			
Red Cell Distribution Width (RDW) in %	19.6 (5.9)	25.4 (8.2)	.012
Platelets per microliter of blood	232,288 (123,177)	208, 166 (106,554)	.433
Prothrombin time in seconds	20.8 (7.4)	19.7 (8.2)	.620
Partial Thromboplastin Time (PTT) in seconds	60.5 (30.9)	46.5 (16.4)	.039
International Normalized ratio (INR)	1.5 (0.4)	1.5 (0.6)	.815

## Discussion

Our study showed a high prevalence of hematological and coagulation derangements in this group of untreated children and adolescents with cyanotic CHDs. It is likely that this is an underestimate of the true prevalence since cyanotic CHDs carry very poor 1-year and 5-year survival rates. There was variable implementation of iron supplementation and therapeutic phlebotomy as management strategies, highlighting the need to sensitize practitioners on these simple and affordable interventions that may improve the quality of life their patients. Such interventions include attention to their hematologic and coagulation profiles and interventions that delay or prevent such complications. As children with CHDs in sub-Saharan settings are generally cared for by non-cardiologist practitioners, the creation of awareness and formulation of practical guidelines may help to mitigate the risk of life-threatening complications.

In cyanotic CHDs, uninhibited erythropoietin secretion triggered by chronic hypoxemia, leads to uninhibited erythrocyte production irrespective of depletion of iron stores in a bid to increase oxygen-carrying capacity (22,23). The erythrocytes produced under such conditions are usually iron-deficient, leading to “anemia in the face of polycythemia”(3,11). But this iron deficiency is often overlooked by practitioners (23). Such erythrocytes increase the risk of CVAs and chronic end-organ damage due to intravascular sludging (11).

Simple measures like iron supplementation may significantly decrease these catastrophic risks. As is seen in this study, it is likely that this measure is not uniformly practiced by practitioners. Some patients were not offered iron treatment even after undergoing therapeutic phlebotomy. The practice of performing therapeutic phlebotomy in the late presenter with a very high hematocrit may actually be counter-productive as it increases the risk of iron deficiency anemia, which in turn increases vascular sludging and the risk of CVA and chronic end-organ damage (24,25). Lowering the hematocrit may not always mean lowering the risk of sludging but rather may mean increasing it through microcytosis and increased whole blood viscosity (26). Under such circumstances, correcting iron deficiency anemia and dehydration should take precedence (27). Other suggested managements like hydroxyurea treatment may also be explored (26).

Thrombocytopenia was common in our study as has been demonstrated in previous reports (5,8,9,28). The suggested mechanisms of thrombocytopenia in these populations may include increased destruction of platelets, platelet activation, shortened lifespan of platelets, lack of fragmentation of megakaryocytes in the lungs, depressed bone marrow production of platelets, and others. Our study also showed that there is a negative correlation between the level of hematocrit and the platelet count, as this also been shown by other studies (7,28,29). This is important because measures targeted against a high hematocrit/hemoglobin may help prevent the occurrence of clinically significant thrombocytopenia. Beyond thrombocytopenia, our study demonstrated that there were other abnormalities with coagulation profiles like PT, PTT, and INR. Previous studies have also shown that there are numerous issues with coagulation in such patients including not only low platelet number but also defective platelet aggregation, vascular injury, and dysfunction, endothelial dysfunction, arterial stiffening, and increased D-dimer levels (10,12,13,31). These patients are prone to both hypercoagulability and bleeding. Simple alteration of coagulation profiles may not accurately predict either event due to the complex dynamics influencing thrombosis and bleeding (6).

Regarding classification and comparison by pulmonary blood flow physiology, the majority of patients had decreased pulmonary blood flow. This study did not collect data on birth prevalence of congenital heart diseases. As a result, there could be an important survival bias as patients with cyanotic lesions and increased pulmonary blood flow are more likely to die early due to early congestive heart failure and pulmonary obstructive vascular disease. Decreased pulmonary blood flow was significantly associated with differences in MCH, MCHC, and RDW. However, the number of patients in the reference group (increased pulmonary blood flow) was small, which may have limited our ability to detect other differences. Other important limitations to this study included a small sample size, which may have limited power to detect significant differences and biased results toward the null hypothesis. We were also limited by the number of laboratory parameters we were able to collect; for instance, we were unable to measure plasma ferritin and transferrin levels, which are important metrics of iron storage and utilization.

In conclusion, our study showed a high burden of hematologic and coagulation derangements among children and adolescents with untreated cyanotic CHD. There was variable implementation of iron supplementation and therapeutic phlebotomy as management strategies, highlighting the need to optimize supportive management in this population to mitigate the risk of life-threatening complications. Future studies may consider looking at different healthcare settings within sub-Saharan Africa and include perspectives into the knowledge, attitude, and practice of non-cardiologist physicians caring for this group of patients. It may also be worth looking into the impact of in-service trainings and written guidelines in improving and harmonizing the care for this vulnerable population.
